# Performance Evaluation of the Highway Radar Occupancy Grid

**DOI:** 10.3390/s21062177

**Published:** 2021-03-20

**Authors:** Jakub Porębski, Krzysztof Kogut

**Affiliations:** 1 Faculty of Electrical Engineering, Automatics, Computer Science and Biomedical Engineering, Department of Automatic Control and Robotics, AGH University of Science and Technology, al. Mickiewicza 30, 30-059 Cracow, Poland; 2 Aptiv Services Poland S.A., ul. Podgórki Tynieckie 2, 30-399 Kraków, Poland; krzysztof.kogut@aptiv.com

**Keywords:** occupancy grid, fusion, automotive, perception, evaluation

## Abstract

The quality of environmental perception is crucial for automated vehicle capabilities. In order to ensure the required accuracy, the occupancy grid mapping algorithm is often utilised to fuse data from multiple sensors. This paper focuses on the radar-based occupancy grid for highway applications and describes how to measure effectively the quality of the occupancy map. The evaluation was performed using the novel grid pole-like object analysis method. The proposed assessment is versatile and can be applied without detailed ground truth information. The evaluation was tested with a simulation and real vehicle experiments on the highway.

## 1. Introduction

Almost all automotive perception systems require a stationary representation of the environment around the vehicle. One of the most popular modelling frameworks is the occupancy grid map [1,2]. The occupancy grid is a multi-dimensional spatial lattice, where each cell stands for an independent portion of space [3]. The task of this environmental model is to calculate the probability that each grid cell is either occupied or free based on sensor observations.

In the automotive perception software stack, the occupancy grid is an intermediate step, which combines and accumulates sensor information over time and applies different methods to filter noise. Based on the output grid, components such as localisation [4,5], path-planning [6] and scene recognition [7,8] make decisions about future vehicle actions.

Both the development of the occupancy grid algorithm and the decision-making process require a measure of quality for the computed grid maps. The evaluation of a map is currently often carried out by visual inspection, leading to highly subjective results [9,10]. Another evaluation method for the occupancy grids is using cell-wise binary comparisons with the reference maps [11,12,13]. This approach is useful in grid development; nonetheless, it already contains a decision-making binarisation process, which may alter the results of the assessment.

The third type of occupancy grid evaluation technique is the detection and monitoring of specific grid object representations. This category of quality measurement is suited for specific grid applications.

In the literature, there are metrics for object tracking [14] and for collision risk estimation [15,16]. In the object extraction steps of these methods, the occupancy grid must be carefully analysed and compared with accurate scene descriptions. This limits their application to only well-described test scenarios or to precisely labelled experimental data.

The aforementioned methods of occupancy grid evaluation require accurate and dense ground truth. The data collection and labelling will contribute to the overall cost of algorithm verification. This article proposes a novel approach for grid object representation that can be used with a sparse scene description. Moreover, the reference information used in the procedure can be easily obtained from an independent parallel perception setup, thereby limiting the effort required for the implementation of new algorithm versions.

The occupancy grids can be applied to many environments and sensor setups. One possible application is a radar occupancy grid for highway applications.

A highway is a high-speed, multi-lane road with separate carriageways for the two directions of traffic, separated from each other by a dividing strip not intended for traffic. The road does not have any level crossings with any other road, railway track, tramway track or footpath and is reserved for specific categories of road motor vehicles [17]. Such conditions lead to a restricted operational domain of perception systems in terms of exposure to crossing vehicles or animals. This limits the region of perception interests only to the front and rear road areas, which can be defined as a lateral corridor.

Radar sensors are commonly used for the realisation of intelligent transportation systems to monitor, track and manage vehicular traffic on roads, and for the mapping and localisation of vehicles [18,19,20]. In general the radar sensor guarantees a robust performance for vehicle localisation, especially in adverse weather conditions (e.g., rain, snow or fog). The type of radar varies according to the frequency band and the modulation method. Among various radar sensors, in the series automotive applications only small millimetre wave radar sensors are used due to their good performance capabilities and excellent price competitiveness [21].

Despite the above advantages of previous works, radar detections may receive noise from various sources; hence, in order to filter them, a decay method together with free space determination must be involved. Free space modelling extracts information on non-occupied space from sensor detections. It is commonly computed as a ray cast to the detection. The other filtering method—decay—introduces the forgetting factor to the grid map, enabling the efficient limitation of occupancy map overconfidence [22,23].

The main contributions of this work are therefore the design and analysis of an occupancy grid evaluation method; we provide an overview of the filtering capabilities of the algorithm. The proposed assessment method is able to work on sparsely distributed ground truth information, and thus requires little labelling effort in order to compute the map quality factors. Within the article, the proposed metrics are analysed in the context of a simulation and a real-world setting.

Several types of occupancy grid filtering methods are presented in Section 2. In Section 3, key performance indicators (KPI) are proposed and defined. The experimental setup and simulation research methodology are presented in Section 4. The filtering capabilities of the occupancy grid algorithm are examined alongside simulation data in Section 5. The experimental results are described in Section 6. A summary and development possibilities are given in Section 7.

## 2. Related Work

The occupancy grid algorithm commonly deals with heterogeneously noisy detections. Detection positioning uncertainty is a sum of multiple factors, such as different transport delays of measurement messages; host positioning uncertainty; sensor mounting inaccuracy; and finally, the detection accuracy itself. Each of these factors contributes to the final detection representation on the occupancy grid. Moreover, the perception sensors are vulnerable to type I errors (false positives), which results in reporting detections which do not represent any real object.

In order to deal with this noise, the occupancy grid algorithm applies various filtering methods. Firstly, in order to reproduce uncertainties, the algorithms develops different sensor models. Free space determination minimises the impact of single-shot false positives. Finally, the probability decay might be applied to limit the overconfidence of other filtering methods.

### 2.1. Occupancy Grid Framework

**Figure 1:**
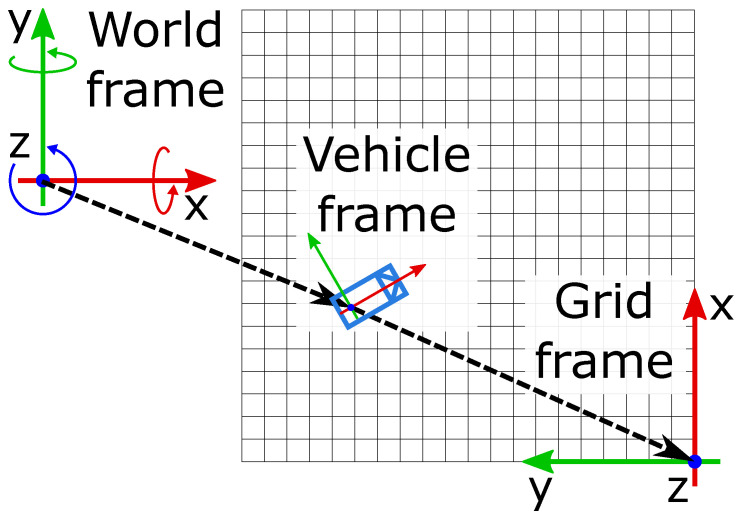
Coordinate systems used to position the occupancy grid in the world frame.

The occupancy grid is a way to represent the environment around a vehicle, so it needs the defined coordinate system of the host; in this case a derivative of the ISO 8855:2011 standard on vehicle dynamics is applied. Following this norm, a right-hand-sided occupancy grid coordinate system can be defined. An example of the occupancy grid is shown in Figure 1.

The environment’s representation on the grid is discrete, although the vehicle is moving and turning in the real, continuous world. In order to eliminate discretisation errors which would occur if the map were to be rotated, the host performs its motions relative to the grid with a fixed orientation [24].

Moreover, since the host may turn on the map, the occupancy grid has to be a square to ensure constant representation of the area in front of the vehicle. This condition implicates that the grid resolution in both dimensions is equal and cells are squares [25].

### 2.2. Sensor Modelling

Each occupancy grid input sensor has unique properties, which can be utilised to extract more information about the environment. The sensor modelling part of the occupancy grid algorithm aims to improve the accumulation procedure of transferring as much information as possible from the detection model to the occupancy grid. The sensor model has to take into account all types of uncertainties present in the occupancy grid. Usually in the experimental setup, many uncertainty characteristics are not directly measurable and the sensor model has to approximate the overall grid detection uncertainty, not only the sensor parameters itself.

The modelling can be performed via forward or inverse sensor modelling. Forward methods optimise the occupancy distribution based on accurate physical models of sensors. This type of modelling requires a lot of computational power and is not considered in the presented evaluation analysis.

The second type of modelling—the inverse sensor model—spreads the detection point into the occupancy probability based on probabilistic distribution. This type of representation is commonly employed in occupancy grid algorithms. Sensor models may be differentiated based on the number of dimensions in which the distribution is computed.

**Figure 2:**
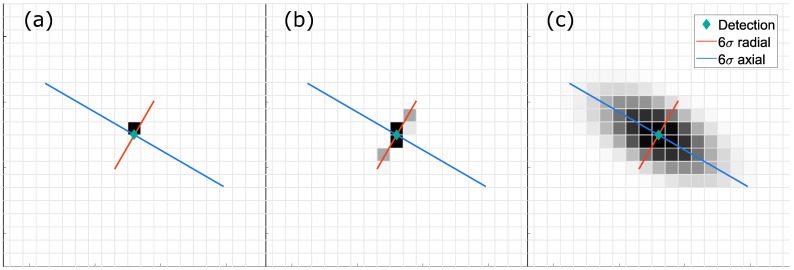
Types of sensor models: (**a**) hit point; (**b**) 1D model along the detection range direction; (**c**) 2D approximation of detection uncertainty. Darker areas represent cells with higher probabilities.

The simplest and most widely utilised sensor model is the zero-dimensional hit-point model (Figure 2a). Its distribution assigns full occupancy probability to the cell where the detection is located, and no evidence to other cells. This model is widely exploited due to its minimal computational and implementation requirements.

One-dimensional models approximate distribution in range or cross-range directions only (see Figure 2b). This type is best suited for the sensors, where most of the occupancy distribution is allocated along the radial or axial lines. It offers medium computational complexity, and the probability values might be computed with exact precision.

Two-dimensional probability models compute the occupancy distribution in Cartesian or polar coordinates around the detection (Figure 2c). The probability value is usually approximated as a Gaussian distribution with the constraint that the sum of all evidence should be equal to the detection’s existence probability [22].

### 2.3. Free Space Determination

Free space modelling exploits the fact that range sensor detections provide information not only about the obstacle but also about empty areas along the space traversed by detection. This determination is developed by casting rays to the detections. The traversed space is then updated to increase the free space probability along the ray.

This type of filtering is extensively utilised for Lidar occupancy grids, where the overall detection density is higher [26]. The ray casting technique in such application leads to Moiré artefacts, or false free space determination.

This article implements the triangle ray casting method presented in prior research [27], which solves the problem of artefacts by using a wider area for updates. Every cell in the triangle ray is filled with the same value, thereby forming a uniform free space probability distribution.

### 2.4. Decay

The third filtering option of the occupancy grid is the decay. It artificially diminishes the evidence on the entire grid over time. The key limitation of sensor and free space modelling is the simplification of many physical dependencies—the algorithm assumes data completion and cell independence. During the process of integrating multiple pieces of evidence, hidden dependencies are omitted. Consequently, the results quickly become overconfident [22]. In order to deal with the problem, the exponential information decaying procedure is applied. This increases the uncertainty of grid cells while preserving the overall variance of the map. The exponential decay is described as:

(1) $$p\left(t+\Delta t\right)=\left(p\left(t\right)-0.5\right)\cdot e^{-\frac{\Delta t}{\tau }}+0.5$$

where p(t) is probability of the cell at time *t*; Δt is the time elapsed between two decay operations; and τ is the mean lifetime, i.e., the time after only ≈36.7% of the evidence remains in the cell.

**Figure 3:**
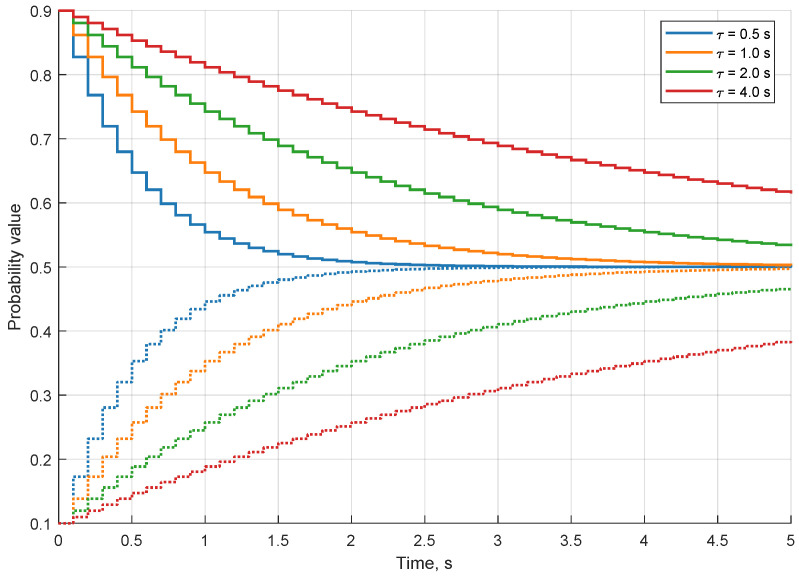
Decaying process of the occupied cell with p=0.9 (solid lines) and free cell p=0.1 (dotted lines) for different mean lifetimes. The decay is applied with 10 Hz frequency.

The behaviour of the decay depends on the update rate of the occupancy grid. The example process of decaying for both occupied and free cells for various τ values is depicted in Figure 3. The decay always tends towards the most uncertain probability value which is 0.5 for the Bayesian inference method.

The overconfidence of occupancy modelling may be handled using different methods, other than the exponential decay mentioned above [22]. Nonetheless, in order to limit the possible parameter space, the presented evaluation analysis covers only the impact of the constant exponential decaying factor on the occupancy grid.

## 3. Definition of Key Performance Indicators (KPI)

The occupancy grid algorithm is a comprehensive solution which can be tuned to the desired application by a plurality of calibration factors, ranging from grid size and resolution, through to different sensor modelling possibilities and decay mean lifetime adjustment. The development and tuning of the algorithm require reliable metrics to measure the quality of the map.

A common approach for the evaluation of a map is visual inspection combined with the algorithm’s expert knowledge. Nonetheless, this strategy often leads to highly subjective results [9,10]. In order to enable the systematic validation of the occupancy map, binary classification KPIs such as false positive rate, precision and recall are applied against the reference ground truth map. Nevertheless, this classification can be performed only when the ground truth is available. Determining complete ground truth for a stationary environment poses major practical challenges. Such metrics can be conveniently applied in the simulated scenarios [13]. Application of continuous classification KPIs, such as a map score and covariance, also does not give satisfactory results [11]. As presented in [11], the existing quality measures from robotics are not adequate for automotive applications. The different goals in robotic and vehicle mapping limit the quality of an automotive map from a robotic viewpoint.

Another approach for the evaluation of a grid map is the extraction of specific object representations and validation of their quality. This approach was first proposed by [10] for robotic applications. The procedure was extended into automotive applications by [15,16] for collision risk estimation and by [14] for grid-based object tracking. Nonetheless, previous evaluation methods are applicable only for specific test drive scenarios.

The proposed assessment method is based on the extraction of specific grid object representations. Landmarks used by the method should be common and easily distinguishable from the surrounding occupancy. On highways, static objects such as barriers, vegetation, signs and others can be captured on the grid if encountered.

**Figure 4:**
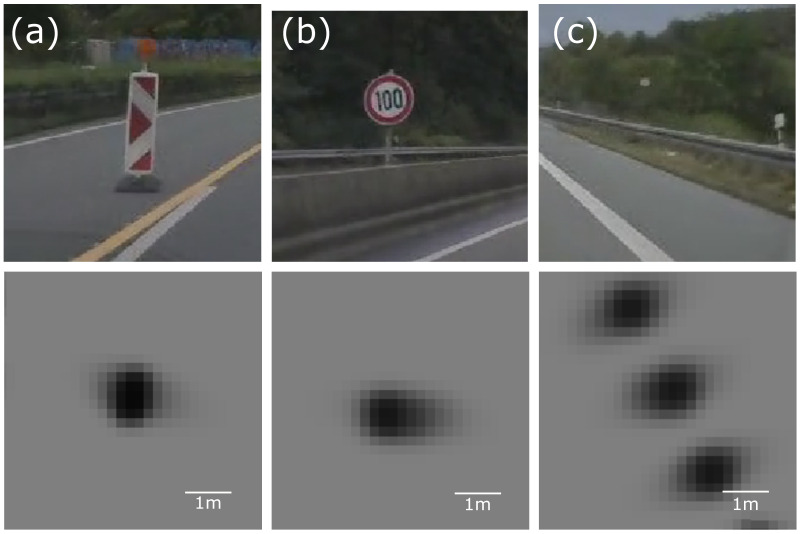
Examples of the occupancy grid representations of highway pole-like objects with corresponding video frames: (**a**) traffic bollard; (**b**) sign; (**c**) guardrail. The occupancy grid is generated using a single automotive-graded radar located in front of the vehicle.

The evaluation criteria are met for signs and guardrails, whose metal poles effectively act as single scattering centres for radar. Those pole-like objects generate circular or elliptical grid map representations, as presented in Figure 4 for signs, bollards or even guardrails. For guardrails (Figure 4c), all the detections are reflected from the poles supporting the guardrail, not from the railing itself, resulting in the characteristic dotted line of occupancy.

Other object representations also might be used as evaluation metrics for the occupancy grid; however, they would require a complex assessment procedure. If the object has a complicated shape (as buildings and vegetation do), the grid representation will be different, based on the object’s angle of incidence. Moreover, arbitrarily shaped objects might be subject to occlusions which make the assessments even harder.

The specifications of a pole and its representation make it an ideal candidate for the evaluation reference point. The pole’s representation will be independent of the vehicle’s relative direction. Furthermore, poles are one of the most common objects in the highway environment. They are also clearly visible for other sensors, such as Lidars, which makes the reference mapping easier.

The grid representation of a pole should preserve the number of objects from the real world, limit their variance and maintain the object’s shape. In order to meet these requirements, the pole-like object’s representation must be compact, circular and occupy the finest space. These attributes are the basis of a KPI definition.

### 3.1. Compactness

Compactness describes whether the pole’s image on the occupancy grid can be expressed as one solid and convex object. Compactness measures the density of a representation and in image processing may be called the solidity of an object. A measure of compactness can be obtained as a ratio of the object’s area to the area of a convex hull of the object (2).

(2) $$\mathrm{Compactness}=\frac{\mathrm{Area}}{\mathrm{Convex}\;\mathrm{area}}$$

where Area is the number of occupied cells classified for the object and Convexarea is the number of cells which lie within the convex contour around the selected occupied cells.

**Figure 5:**
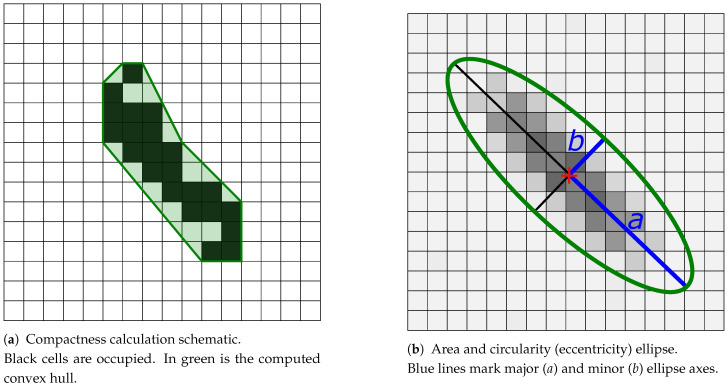
Schematic visualisation of key performance indicators (KPI).

For evaluation purposes, every updated cell is taken into account; the convex hull size is computed over the measured pole representation area. Visualisation of this KPI measure is presented in Figure 5a.

If the compactness of the representation is close to zero, the object is sparse. In this case, it might be separated into multiple objects by clustering or contour algorithms. The best value of compactness should be as close to 1 as possible. This KPI must use some arbitrary threshold to identify the cell cluster and to compute compactness. Computation of this KPI value is a validation check enabling further quality assessment.

### 3.2. Area of Object Representation

KPI compactness measures an object’s area as a number of classified cells. Such area approximation, however, does not consider individual cell probabilities and can be used only for rough object validation and not for the estimation of the object’s variance.

The pole-like object representation on the occupancy grid plane is generated by radar scattering centres located in the pole’s section. Hence, the grid object may be approximated as a Gaussian distribution. The estimated pole variance is measured as the area of distribution stretching over the σ range.

The mean value of the Gaussian distribution is computed as a weighted centroid (3) taking into consideration cell probabilities.

(3) $$\mu_{w}=\frac{\sum_{i=1}^{N}\left(w_{i}\cdot x_{i}\right)}{\sum_{i=1}^{N}\left(w_{i}\right)}$$

where:

*N*—number of selected object’s cells;
$w_{i}$—i-th cell probability;
$x_{i}$—i-th cell coordinate.

Having a calculated mean value, the covariance matrix may be obtained. For area and further circularity measurement, however, only the information about distribution eigenvectors is required. Eigenvectors might be computed as weighted standard deviation values in orthogonal directions (4). The angle of distribution orientation is found by numerical optimisation.

(4) $$\sigma_{w}=\sqrt{\frac{\sum_{i=1}^{N}(w_{i}\cdot {\left(x_{i}-\mu_{w}\right)}^{2})}{\frac{\left(M-1\right)}{M}\sum_{i=1}^{N}\left(w_{i}\right)}}$$

where *M* is the number of cells with non-zero weights.

The approximated distribution area for given standard deviations is an ellipse with major and minor axes equal to computed values (Figure 5b). The variance represents an area of the ellipse stretching over the σ range (5).

(5) $$\mathrm{Area}\;\mathrm{of}\;\mathrm{occupancy}=\pi \cdot \sigma_{a}\cdot \sigma_{b}$$

where $\sigma_{a}$ is the standard deviation over the semi-major axis of the fitted distribution, and $\sigma_{b}$ is the standard deviation over the semi-minor axis. In order to minimise the pole-like object representation spread, the variance value should be minimised as well. The KPI value represents the area within 1σ level set representing only 68.27% of the object. In order to cover at least 95.45%, the value should be multiplied by 4.

### 3.3. Circularity

The circularity factor describes how well the occupancy grid algorithm preserves the shape of the pole-like object. The third KPI value is computed by measuring the approximated Gaussian distribution’s eccentricity (6).

(6) $$\mathrm{Circularity}=\sqrt{1-\frac{\sigma_{b}^{2}}{\sigma_{a}^{2}}}$$

The perfect circle eccentricity value is 0; thus, the circularity should be minimised to get the best grid map quality.

## 4. Experimental Setup

The highway environment triggers a restricted set of use cases of automotive perception. This type of road usually provides lack of cross traffic, good accessibility of HD maps and accurate GPS positioning available in the open sky [17]. All these features may simplify the automated vehicle system to perceive only the road corridor.

The occupancy grid depicts the entire surrounding area of the vehicle, so the KPI values should not be measured in one point. As the grid accumulates information, the best accuracy is expected near to the host or for the obstacles already passed by the host. Depending on the algorithm’s application, various parts of the map have distinct evaluation priorities. For example, the front part of the map is crucial for planning modules to assess the correct road situation.

In the highway environment, the area with short lateral distances from the vehicle contains most of the relevant occupancy information. The evaluation focuses on the front sensing segment of the map and analyses how the quality changes with variable longitudinal distance from the vehicle.

The occupancy grid algorithm implementation was developed based on [23,28]. Grid map size was selected as 150×150 m with a cell resolution of 0.2 m based on previous highway grid parameter reviews [25]. The sensor models were designed using dual architecture, as presented in [27]. The occupancy grid vehicle was moved to the rear border to ensure a minimum of 100 m of mapping in front of the vehicle.

The occupancy grid algorithm quality depends on multitude of parameters. To verify whether the proposed assessment method is correct, the number of tunable criteria have to be minimised.

The vehicle position and motion characteristics’ uncertainties have an impact on the occupancy grid performance. To simplify error analysis, it is assumed that the host position is known and accurate. This condition is trivial for simulations; however, to maintain it also in the real world experiments, a high precision global positioning system (GPS) and an inertial measurement unit (IMU) are used for the vehicle localisation.

In the automated vehicle, multiple radars were mounted to ensure full coverage around the vehicle. Nevertheless, this article focuses only on the area in front of the vehicle, thereby limiting the sensor set to only front sensor and simplifying the calibration set. Besides that, the evaluation procedure might be applied also on the multi-radar occupancy grid with the consideration of the synchronisation uncertainty.

The radar sensor used was an automotive grade frequency-modulated continuous-wave radar sensor operating in millimetre-wave bandwidth. Radars available in the commercial automotive market operate with around 50 ms update rates and produce on average 60–150 detections (point targets) from the sensed field of view [29].

### 4.1. Simulation

In order to test the performance of the evaluation method under strictly controlled conditions, a simulated scenario has been prepared.

**Figure 6:**
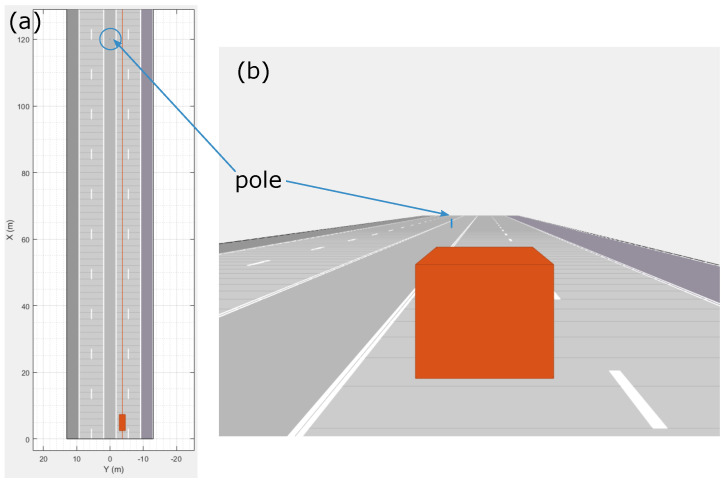
Highway simulation with a single pole: (**a**) bird’s-eye view; (**b**) ego-centric view.

The simulation has been designed using the *Driving Scenario Designer* toolbox available in Mathworks MATLAB. The structure of the simulation was simplified to a minimum to limit the calibration parameters and to optimise evaluation time. It mimicked the highway scenario conditions: the host moved with a velocity of 30 m/s, which corresponds to 108 km/h, on a straight road. The only obstacles in the scenario were rectangular poles with dimensions of 10×10×100 cm. One of these obstacles in the simulation view is presented in Figure 6.

For testing purposes, the simulated vehicle was equipped with an ideal range sensor with a $360^{\circ }$ field of view. As the grid size was 150 m, the pole was present on the grid for around 5 s (based on host velocity). The common radar sensor generated a detection scan every 50 ms and the pole of size 10×10×100 cm was represented by 1–2 detections in the scan. This means that a maximum of 100 detections can be collected and stored on the occupancy grid from one sensor when driving near the pole.

### 4.2. Real World Experiments

The experimental setup consisted of an automotive grade radar mounted on the front bumper of the vehicle. The standard deviation of the reported detection’s position was taken to be $\sigma_{r}=0.3$ m for range and $\sigma_{\phi }=1^{\circ }$ for azimuth.

The experimental data were collected on a highway where the speed limit ranged from 100–130 km/h. The vehicle position and motion parameters were obtained using high precision GPS and an IMU unit. For evaluation purposes, small pole-like objects were extracted from the occupancy grid by comparing the camera-captured images and a satellite map of the area. For identified poles, KPIs were calculated. The results of the evaluation are presented in Section 6.

## 5. Simulation Results

**Figure 7:**
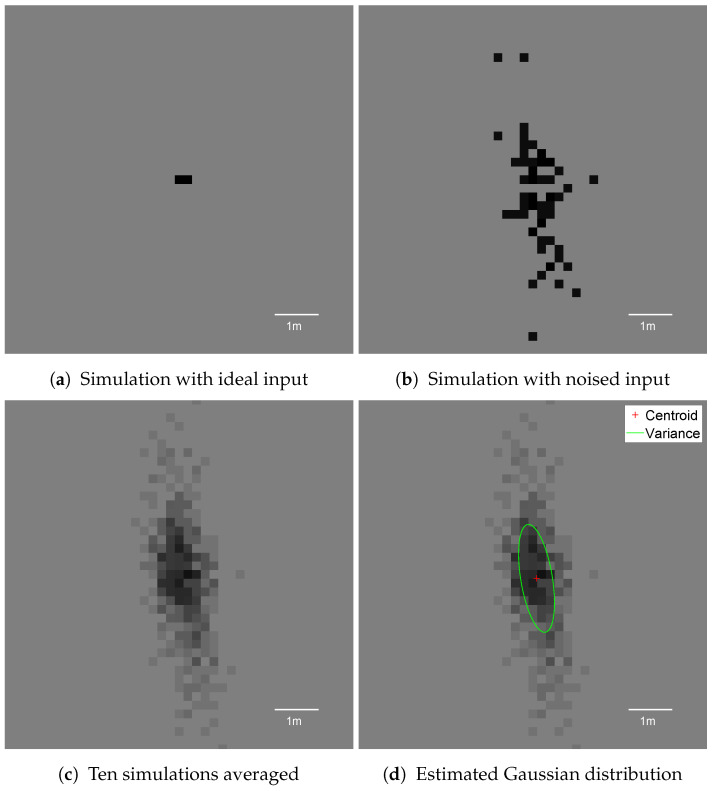
Occupancy grid representations of the pole using a simple hit point sensor model with disabled filtering methods. Each snapshot presents the pole located laterally 10 m to the right side of the vehicle, captured when the host was 10 m longitudinally from the pole.

The ideal pole object after accumulation occupied up to two cells on the occupancy grid (see Figure 7a). The generated ideal detections were used to simulate different noise conditions with variance of the experimental sensor ($\sigma_{r}=0.3$ m and $\sigma_{\varphi }=1^{\circ }$). In order to imitate false positive readings, the confidence level of detections was lowered to 90%, meaning that one detection could produce at least 0.9 occupancy evidence probability. Due to the application of random detection spread on only around 100 detections, a single simulation run might generate sparse distribution of the pole on the occupancy grid (Figure 7b). In order to visualise the distribution of occupancy grid representation, the simulations were repeated 10 times, and averaged results are depicted in Figure 7c. On top of the accumulated data, the centroid and variance are presented in Figure 7d.

### 5.1. Characteristics of the Occupancy Grid Data Accumulation

An occupancy grid accumulates incoming sensor data over time to compute the most reliable estimate of the environment. The accumulation itself, however, may lead to overconfidence of estimates and noise amplification. The accumulation only approach is useful solely for noise level estimation and is used to verify how the defined key performance indicators behave in different conditions.

The first experiments performed on the grid included disabled decay and free space modelling, which uses a 0D (hit point) sensor model. The output pole images are presented in Figure 7.

As the host moved towards the test pole, more data became available. The KPIs were measured as a function of the longitudinal distance to the vehicle, highlighting the data accumulation process.

**Figure 8:**
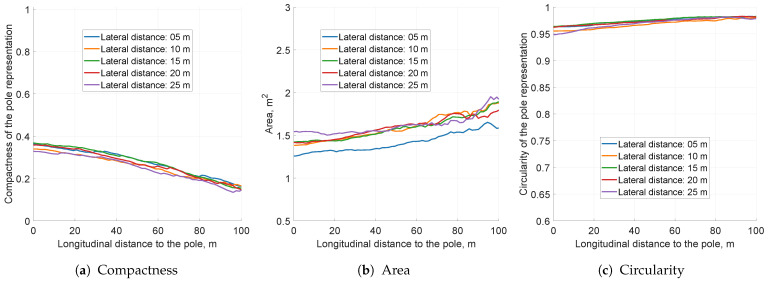
The key performance indicators computed for the accumulation-only grid configuration. The scaling of this figure is the same for all ensuing simulation KPI plots, for easier value comparison.

The defined key performance indicators were first tested with several lateral pole locations. This simulation aimed to measure whether the assessment can be applied to a variety of pole-like object positions in the environment. The results presented in Figure 8 show similar behaviour for all lateral positions. The pole offsets were tested up to 25 m laterally because most of the relevant occupancy grid information in the evaluated highway scenario was limited to a ±25 m lateral corridor. This means that the presented framework can be utilised for a range of lateral positions and the results will be comparable to each other.

The analysis of grid performance starts with the compactness metric. The value ranges from 0.15 to 0.35, which means that the raw object representation is not compact (see Figure 8a). The variance originating from noisy detections ($\sigma_{r}=0.3$ m and $\sigma_{\varphi }=1^{\circ }$) results in a representation surface of 1.5–2 m${}^{2}$. The result generated by ideal detections had a variance of around 0.3 m${}^{2}$, which matches the grid discretisation error. The circularity of the ideal object was estimated at 0.87 due to two-cell representation. Even without any filtering, the estimated object variance slightly reduced closer to the vehicle, due to the fact that as the range decreased, the azimuth noise declined and the object shrunk (Figure 8b). For all tested pole positions, the circularity KPI was close to 1, which means that the object was far from a circular shape (Figure 8c).

Various tested pole lateral positions did not show significant differences in KPI values. As a representative example, the pole located 10 m laterally from the left side of the vehicle was selected for further evaluation.

The following tuning procedure successively applied more filtering methods to observe changes in the KPI values.

### 5.2. Impact of the Sensor Modelling Type on the Pole Representation

**Figure 9:**
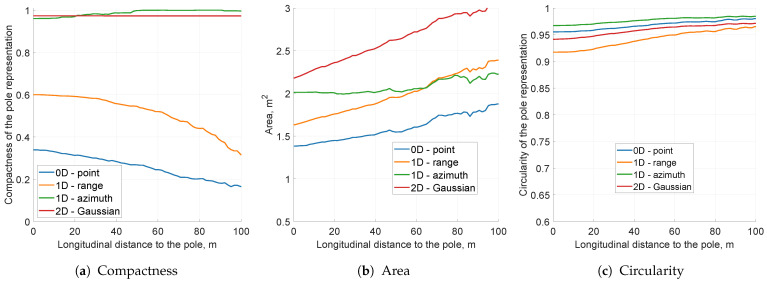
The key performance indicators computed for the various sensor modelling approaches for the pole moving 10 m laterally at a time. The scaling of this figure is the same as that of all other simulation KPI plots.

The simulation experiments were performed on the same data as previous runs with the sensor model configured to mimic sensor uncertainty values ($\sigma_{r}=0.3$, $\sigma_{\varphi }=1^{\circ }$). The KPI values represented as a function of a longitudinal distance to the vehicle are presented in Figure 9.

**Figure 10:**
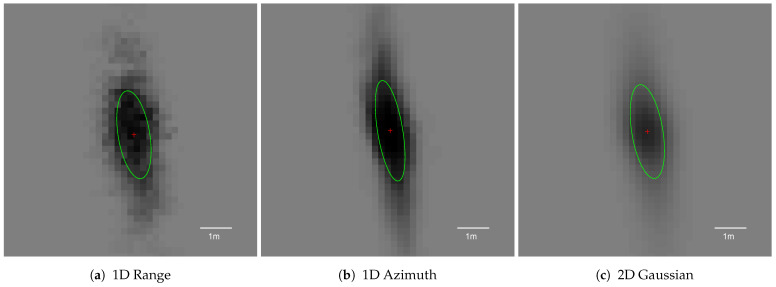
Averaged occupancy grid representations of the pole using different sensor models. Each snapshot presents the pole located 10 m from the left side of the vehicle, captured when the host was 10 m away longitudinally.

Regarding grid snapshots for distinct types of modelling techniques (Figure 10), the area of object representation increased significantly, which is also visible in Figure 9b. This was an expected behaviour, because further filtering methods handle an indistinct pole representation only if the pole image is compact. As presented in Figure 9a, the solidity of the grid object was high for both 1D azimuth and 2D sensor models. Circularity values for each model were still close to 1 meaning that the objects were far from circular (see Figure 9c).

For the radar with specified uncertainty characteristics, both 1D azimuth and 2D sensor models may be applied. Nevertheless, comparing 1D azimuth and 2D Gaussian grid snapshots, the 2D sensor model delivered smoother results (Figure 10b,c. More complex modelling utilised for the two-dimensional distribution can encode more information in cell occupancy probabilities and may yield better results after further filtering steps. Based on that observation, for the next parameter’s evaluation, the 2D probability model was selected.

### 5.3. Impact of the Free Space Determination on the Pole Representation

Free space modelling is intended to eliminate mostly false positives and sharpen the vehicle’s immediate surroundings. The free space gain factor is defined as the maximum value of free space probability assigned for cells updated in the free space determination process.

**Figure 11:**
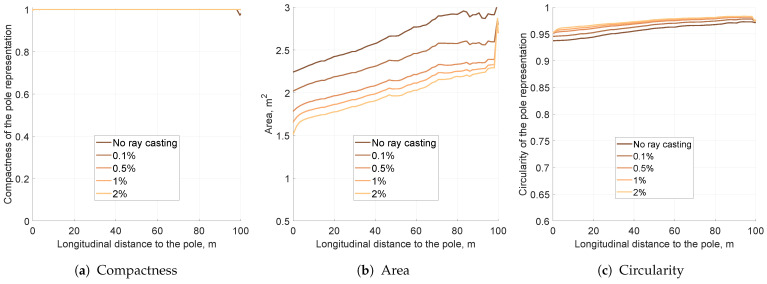
The key performance indicators computed for the set of lower free space probability gains for the pole moved by 10 m laterally per step with the application of a 2D Gaussian sensor model. The scaling of this figure is the same as that of all other simulation KPI plots.

**Figure 12:**
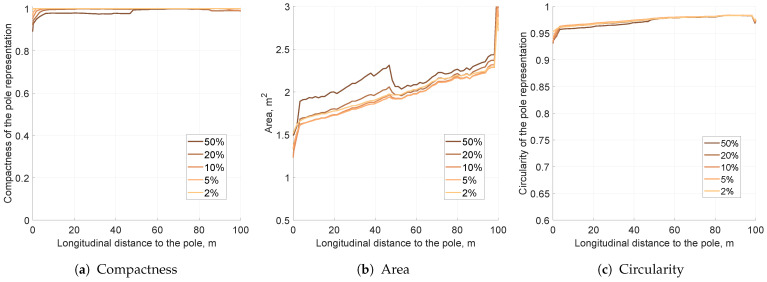
The key performance indicators computed for a set of higher free space probability gains for the pole moved by 10 m laterally per step with the application of a 2D Gaussian sensor model. The scaling of this figure is the same as that of all other simulation KPI plots.

The free space simulation results are presented in Figure 11 and Figure 12. Simulations were launched with a 2D Gaussian sensor model for the pole moved by 10 m laterally. The free space gain factor is represented in percent, which denotes how much of the evidence was introduced for every ray cast. For example, a 2% gain means that in every fusion step the probability of 0.49 was fused into the cell (7).

(7) p(2%)=0.5−0.5·2%=0.49

The KPI plots are split into two sections: Figure 11 depicts lower free space gains, while Figure 12 represents higher gain values. For all tested free space factors, the compactness value was close to 1, meaning that the object’s solidity did not degrade due to ray casting (Figure 11a and Figure 12b). The circularity value slightly increased with growing free space gain, but the change was negligible (Figure 11c and Figure 12c).

**Figure 13:**
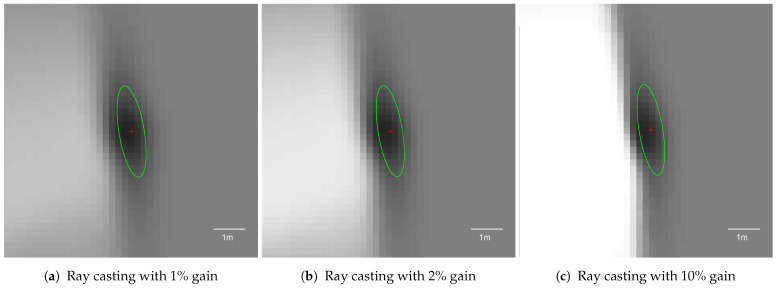
Averaged occupancy grid representations of the pole using different free space evidence gains with a 2D Gaussian sensor model. Each snapshot presents the pole located 10 m from the left side of the vehicle, captured when the host was 10 m awawy longitudinally.

The main impact of free space determination is visible in the variance KPI measure. For the smaller free space gain, the estimated object area contracted (Figure 11b). The higher free space gains, however, resulted in increasing object variance (Figure 12b). For high-gain free space, even a single ray may disrupt the occupancy estimation and decrease the peak value significantly, as presented in the snapshot in Figure 13c.

The estimated minimum variance of the object was observed between 2 and 5% free space gains. The lower and higher gains resulted in larger pole representations, as presented in the snapshots in Figure 13. As the optimal for the further evaluation, 2% gain was selected.

### 5.4. Impact of the Decay Factor on the Pole Representation

The simulations for the third evaluated filtering method—decay—were performed similarly to previous ones. The decay factor is driven by the mean lifetime (λ) value. It is estimated that the larger the λ, the slower the decay, which decreases probabilities.

**Figure 14:**
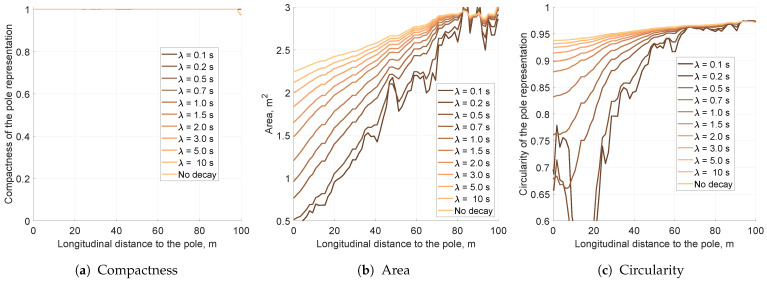
The key performance indicators computed for the different values of decay mean lifetime for the pole moved by 10 m laterally.

The results for different decay factors show some interesting traits (see Figure 14). Independently of decay mean lifetime, compactness was always preserved and remained high (Figure 14a). As decay sped up, the area of the pole representation decreased (Figure 14b). Decay, however, was only applied to decrease fusion overconfidence, and higher values may result in object placement instability. This could be observed for λ={0.1s,0.2s,0.5s}: the area plots are no longer monotonic with increasing longitudinal distance.

**Figure 15:**
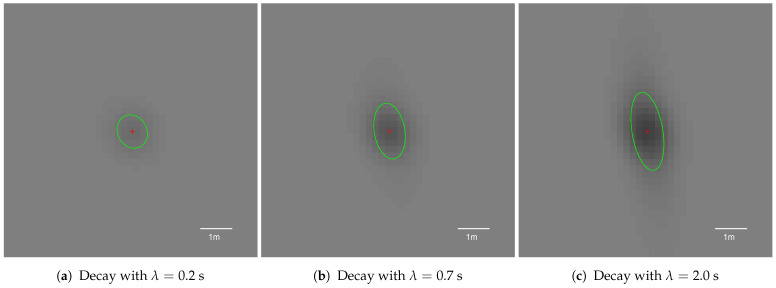
Averaged occupancy grid representations of the pole using different decay mean lifetimes with a 2D Gaussian sensor model. Each snapshot presents the pole located 10 m from the left side of the vehicle, captured when the host was 10 m away longitudinally.

Decay also decreases object representation eccentricity, leading to a significantly improved circularity factor (Figure 14c). Nonetheless, for low mean lifetimes of λ={0.1s,0.2s}, circularity plummeted to a value of 0.4 at a distance of 15 m from the vehicle. The object snapshot became circular, as presented in Figure 15a; however, almost all occupancy evidence was eradicated. This means that too fast of a decay may lead to the removal of significant grid evidence.

Slower decay factors λ≥0.7s also decrease the object representation area and circularity, but in a more subdued manner. The KPI plots for those values are monotonous, meaning that decay does not remove vital object evidence. The grid pole representation snapshots are presented in Figure 15.

As the optimal decay for that grid setup, a value of λ=0.7 was selected. This value offers fair area and circularity reduction. The impact of the decay factor is still significant; however, the output object is compact and can be easily extracted with minor variance, as presented in Figure 15b.

### 5.5. Combination of Filtering Methods

**Figure 16:**
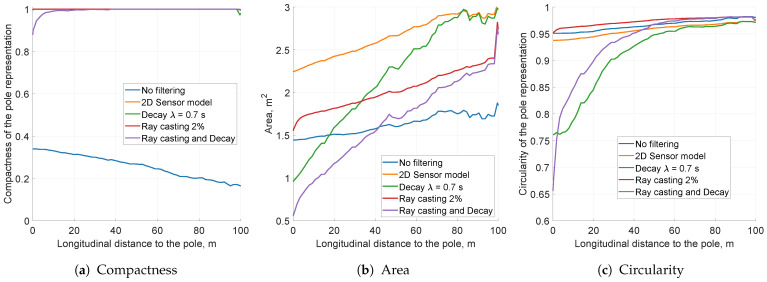
The key performance indicators computed for the simulation data for different variants.

**Figure 17:**
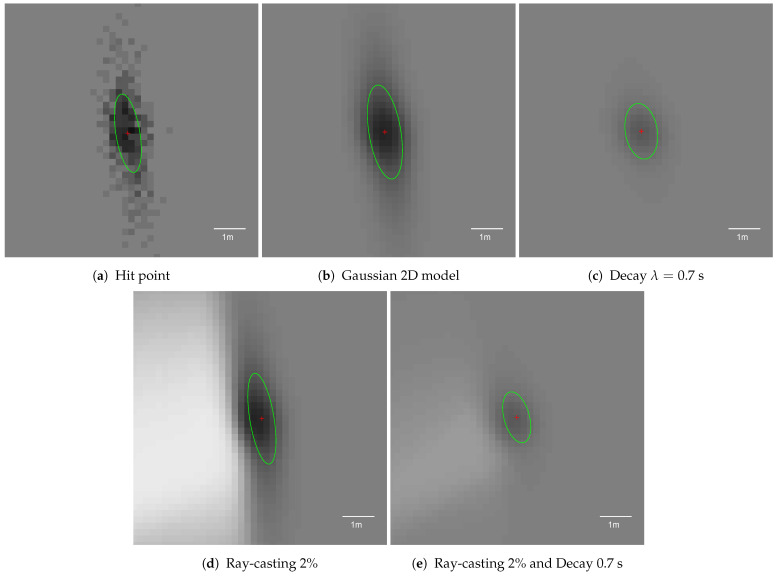
Averaged occupancy grid representations of the pole using different filtering methods. Each snapshot presents the pole located 10 m from the left side of the vehicle, captured when the host was 10 m away from the pole longitudinally.

Having analysed the impact of each filtering method, the final results may be presented. Figure 16 and Figure 17 depict the simulated KPI values and snapshots of each selected method’s parameters.

The unfiltered data presented in the snapshot in Figure 17a are characterised by low compactness of the pole object representation. Visual analysis of the snapshots between the unfiltered case and the grid with an enabled 2D Gaussian sensor model (Figure 16a,b) shows that the measured variances of those two instances should be comparable. As mentioned before, however, the low solidity of unfiltered data impairs further KPI measures, making them unreliable. The variance and circularity computations are applicable only if the object representation compactness is close to 1.

The application of the 2D Gaussian sensor model makes the pole object representation fully compact, although the estimated variance is high (Figure 17b). In order to lower the discrepancy, the decay and free space determination methods were applied. Ray casting with a gain of 2% decreases the pole area evenly for all longitudinal distances from the vehicle. Conversely, decay with a mean lifetime of λ=0.7 s rapidly lowers variance for shorter ranges. Decay also lowers the circularity value, making the output pole more circular (Figure 17c).

The combination of decay and free space determination yielded the lowest estimated area of the grid object, reaching around 1 m${}^{2}$ at a 10 m distance (Figure 16b,c). The circularity of the grid objects was around 0.85. These numerical results are closest to the ideal object area of 0.4 m${}^{2}$ and its circularity of 0.87.

The application of combined filtering and decay methods lowers the occupancy probability on the resultant grid. Nevertheless, reduced cell confidence does not mean lack of occupancy grid quality. The ray cast and decayed snapshot presented in Figure 17e reaches the occupancy probability value of 0.67. This level of confidence is enough for most decision-making processes. Moreover, computing the occupancy probability over cells within the 1σ range, depicted as an ellipse, gives almost certain occupancy in this area.

## 6. Experimental Evaluation

**Figure 18:**
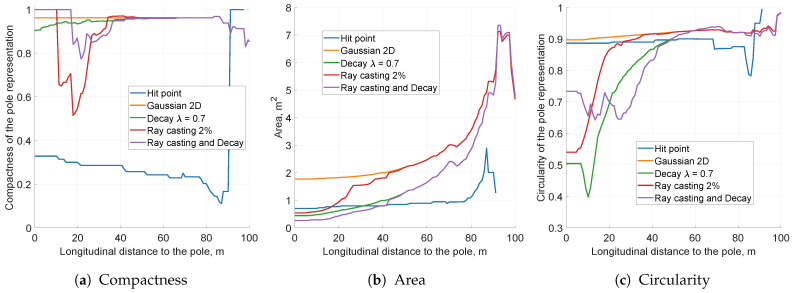
The key performance indicators computed for the experimental data for different variants.

**Figure 19:**
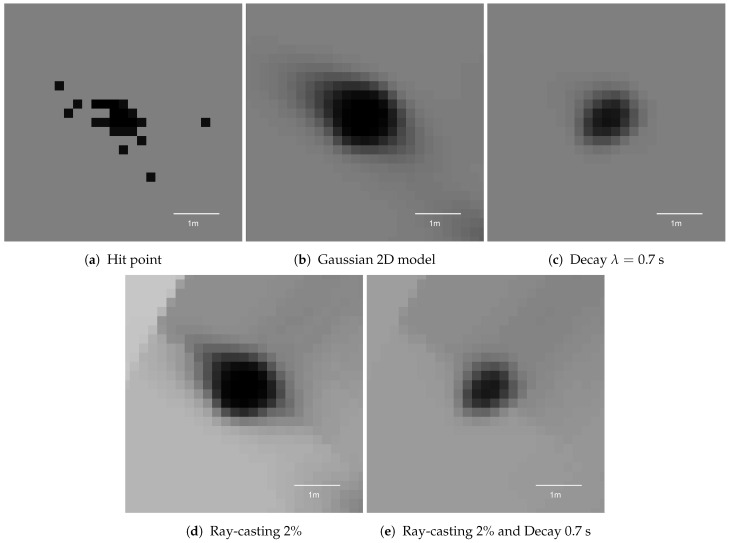
Occupancy grid representation of the pole in the experimental data. The presented snapshots are raw grid data without averaging.

The experimental evaluation was performed based on the parameters tuned in the various re-simulations presented in Section 5. The vehicle drove on a highway with an average velocity of 25 m/s. In this scenario, multiple signs and bollards were selected and evaluated. The measured KPI values from one sample sign are presented in Figure 18. Snapshots of one of the poles for each variant are presented in Figure 19.

The experimental results show similar trends as in the simulation outcomes. The initial hit-point-based occupancy grid exhibits a poor compactness value, invalidating the variance and circularity scores for that variant. As presented in Figure 19a, it should not be estimated as a Gaussian distribution.

The application of a 2D Gaussian sensor model makes the pole appear solid (Figure 19b), but the variance is also high.

The addition of decay filtering decreases the area and makes the pole shape more circular. Conversely, the ray casting itself can reduce the area of the object for closer ranges. The combination of decay and ray casting yields the best results over the largest range of distances. For distances closer than 40 m from the pole, the object representation had an estimated area lower than 1 m${}^{2}$, and circularity reached 0.7 (see Figure 18b,c). These results are close to the ideal object variance of 0.27 m${}^{2}$ and supersede the ideal circularity of 0.87.

**Figure 20:**
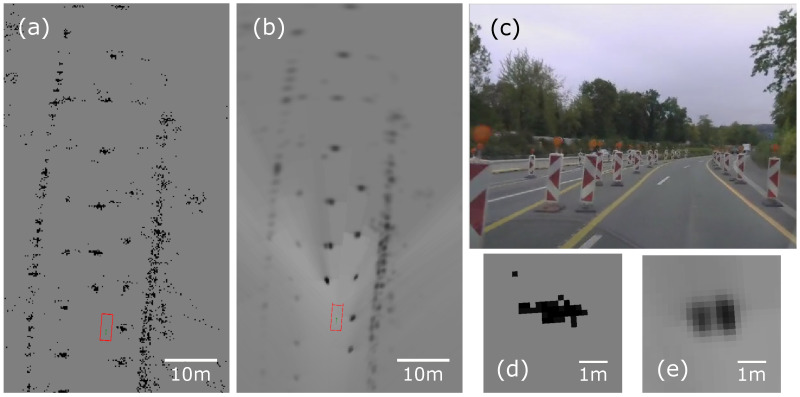
Experimental results for single radar occupancy grids: (**a**,**d**) hit point sensor model without decay and ray casting; (**b**,**e**) decayed and ray-cast grid with the Gaussian 2D sensor model. Image (**c**) shows the vehicle surroundings captured by the in-vehicle reference web camera.

The pole-like objects are not the only obstacles visible on the occupancy grid. A larger part of the grid is depicted in Figure 20. The image presents a screenshot of a short road-works section with high bollard concentration (Figure 20). The image from a web camera serves as a visual reference only. In the initial hit point, occupancy grid detections are scattered in the environment (Figure 20a). The proposed approach with tuned free space and decay shows that false positives in front of the vehicle are filtered out effectively (Figure 20b). Moreover, in the filtered output, two separate bollards close to the vehicle may be separated, which cannot be done using the basic hit point approach (Figure 20d,e).

## 7. Conclusions and Further Work

The occupancy grid algorithm is a powerful perception tool for modelling the environment around a vehicle. Nevertheless, the required mapping quality cannot be achieved without proper tuning (compare Figure 20a,b).

This article proposes a novel evaluation procedure based on pole-like object representation. The landmarks required for the method to run can be easily accessed using other sensors providing information about poles close to the vehicle (e.g., traffic sign recognition from a camera or HD maps). The required accuracy and quality of ground truth information are limited (constrained to object identification on the grid) and do not have to match the grid representation perfectly. Future work might consider evaluation of the grid quality against complex objects if the ground truth for them will be easily accessible.

Both simulation and experimental results show that the main occupancy grid filtering capabilities can be tuned using the proposed framework. The quality was measured by both visual inspection and three different KPI values describing the compactness, variance and shape of the object. The simulation results show that proper sensor modelling ensures the grid object’s compactness. Decay and free space determination help to reduce the overall variance of the estimated objects. From the available parameter space, only selected areas provide a stable occupancy grid. Both simulation and experimental results showed that the lower free space probability and moderate speed decay improved the overall occupancy grid quality.

The presented results are applicable so far to the front region of the occupancy grid (covered by front sensor). The evaluation procedure must be applied to the multi-radar occupancy grid with consideration of the synchronisation uncertainty to determine calibrations valid for the entire grid. The noise generation tool-chain takes into consideration only detection noise with a zero mean property. Other types of uncertainties such as host positioning uncertainty and sensor mounting misalignment should also be tested to determine their influences on the grid map.

The proposed object representation approach to occupancy grid quality assessment is a promising framework. The advocated evaluation method is based on the estimation of continuous occupancy probability and is almost free of decision-making processes. Moreover, it can be applied to any type of grid inference framework, whose representation may be converted into occupancy probabilities, such as those dictated by the Bayesian or Dempster–Shafer evidence theories. The procedure does not require full ground truth knowledge, and object extraction may be performed by other sensors. Several types of extracted objects can be defined for various applications and constitute a tool ensuring the required quality of the occupancy grid mapping method.

## Data Availability

The simulation data presented in this study are openly available in https://github.com/jakpor/grid_performance_evaluation (accessed on 17 January 2021). Experimental evaluation data presented in this study are available on request from the corresponding author. Part of the data is not publicly available due to intellectual property restrictions.

## References

[1] Elfes A., Matthies L. Sensor integration for robot navigation: Combining sonar and stereo range data in a grid-based representataion. Proceedings of the 26th IEEE Conference on Decision and Control.

[2] Moravec H.P. (1988). Sensor fusion in certainty grids for mobile robots. AI Mag..

[3] Dietmayer K.C.J., Reuter S., Nuss D., Winner H., Hakuli S., Lotz F., Singer C. (2016). Representation of Fused Environment Data. Handbook of Driver Assistance Systems.

[4] Stachniss C. (2006). Exploration and mapping with mobile robots. Ph.D. Thesis.

[5] Milstein A. (2008). Occupancy Grid Maps for Localization and Mapping. Motion Planning.

[6] Stepan P., Kulich M., Preucil L. (2005). Robust data fusion with occupancy grid. IEEE Trans. Syst. Man Cybern. Part C (Appl. Rev.).

[7] Erkent O., Wolf C., Laugier C., Gonzalez D.S., Cano V.R. Semantic Grid Estimation with a Hybrid Bayesian and Deep Neural Network Approach. Proceedings of the 2018 IEEE/RSJ International Conference on Intelligent Robots and Systems (IROS).

[8] Seeger C., Muller A., Schwarz L., Manz M. Towards Road Type Classification with Occupancy Grids. Proceedings of the IEEE Intelligent Vehicles Symposium.

[9] Balaguer B., Balakirsky S., Carpin S., Visser A. (2009). Evaluating Maps Produced by Urban Search and Rescue Robots: Lessons Learned from RoboCup. Auton. Robot..

[10] Wagan A.I., Godil A., Li X. (2008). Map Quality Assessment. Proceedings of the 8th Workshop on Performance Metrics for Intelligent Systems—PerMIS ’08.

[11] Grewe R., Komar M., Hohm A., Lueke S., Winner H. Evaluation Method and Results for the Accuracy of an Automotive Occupancy Grid. Proceedings of the 2012 IEEE International Conference on Vehicular Electronics and Safety (ICVES 2012).

[12] Skruch P., Długosz R., Kogut K., Markiewicz P., Sasin D., Różewicz M. The Simulation Strategy and Its Realization in the Development Process of Active Safety and Advanced Driver Assistance Systems. Proceedings of the SAE 2015 World Congress & Exhibition.

[13] Markiewicz P., Porębski J. (2020). Developing Occupancy Grid with Automotive Simulation Environment. Appl. Sci..

[14] Steyer S., Lenk C., Kellner D., Tanzmeister G., Wollherr D. (2019). Grid-Based Object Tracking With Nonlinear Dynamic State and Shape Estimation. IEEE Trans. Intell. Transp. Syst..

[15] Laconte J., Debain C., Chapuis R., Pomerleau F., Aufrère R. (2019). Lambda-Field: A Continuous Counterpart of the Bayesian Occupancy Grid for Risk Assessment. arXiv.

[16] Ledent P., Paigwar A., Renzaglia A., Mateescu R., Laugier C. Formal Validation of Probabilistic Collision Risk Estimation for Autonomous Driving. Proceedings of the CIS-RAM 2019–9th IEEE International Conference on Cybernetics and Intelligent Systems (CIS) Robotics, Automation and Mechatronics (RAM).

[17] Commision E. (2018). Motorways 2018. Technical Report, European Road Safety Observatory. https://ec.europa.eu/transport/road_safety/sites/roadsafety/files/pdf/ersosynthesis2018-motorways.pdf.

[18] Steyer S., Tanzmeister G., Wollherr D. Object Tracking Based on Evidential Dynamic Occupancy Grids in Urban Environments. Proceedings of the 2017 IEEE Intelligent Vehicles Symposium (IV).

[19] Kaul P., De Martini D., Gadd M., Newman P. (2020). RSS-Net: Weakly-Supervised Multi-Class Semantic Segmentation with FMCW Radar. arXiv.

[20] Hong Z., Petillot Y., Wang S. (2020). RadarSLAM: Radar Based Large-Scale SLAM in All Weathers. arXiv.

[21] Stanislas L., Peynot T. Characterisation of the Delphi Electronically Scanning Radar for Robotics Applications. Proceedings of the Australasian Conference on Robotics and Automation.

[22] Thrun S., Burgard W., Fox D. (2005). Probabilistic Robotics.

[23] Gálvez del Postigo Fernández C. (2015). Grid-Based Multi-Sensor Fusion for On-Road Obstacle Detection: Application to Autonomous Driving. Ph.D. Thesis.

[24] Weiss T., Schiele B., Dietmayer K. Robust driving path detection in urban and highway scenarios using a laser scanner and online occupancy grids. Proceedings of the 2007 IEEE Intelligent Vehicles Symposium.

[25] Porębski J., Kogut K., Markiewicz P., Skruch P. (2019). Occupancy grid for static environment perception in series automotive applications. IFAC-PapersOnLine.

[26] Yguel M., Aycard O., Laugier C. (2008). Efficient GPU-based construction of occupancy grids using several laser range-finders. Int. J. Veh. Auton. Syst..

[27] Porębski J. (2020). Customizable Inverse Sensor Model for Bayesian and Dempster-Shafer Occupancy Grid Frameworks. Advanced, Contemporary Control.

[28] Markiewicz P., Kogut K., Różewicz M. Occupancy Grid Fusion Prototyping Using Automotive Virtual Validation Environment. Proceedings of the 6th International Conference on Control, Mechatronics and Automation.

[29] Stuff A. (2021). Radar Comparison Chart. https://autonomoustuff.com/radar-chart.

